# Supplementary material for: Associations between Toxoplasma gondii seropositivity and psychopathological manifestations in schizophrenic patients: A single-center study from Ecuador
Source: PLoS One. 2024 Feb 23;19(2):e0297523. doi: 10.1371/journal.pone.0297523 (PMC10890741; doi:10.1371/journal.pone.0297523)
Supplement: Attachment — Submitted filename: Cover Letter and Response to Reviewers and Editor (Clone).docx [file pone.0297523.s001.docx]

**Date**: December 28^th^, 2023

**To**: Dr. Masoud Foroutan, Academic Editor, PLOS ONE

**From**: Dr. Diego Rosado

**Subject**: Rebuttal Letter - PLOS ONE Decision: Revision Required [PONE-D-23-33384]

Dear Dr. Foroutan,

I am writing to address the decision and the feedback provided for our manuscript, PONE-D-23-33384, titled "Associations between Toxoplasma gondii Seropositivity and Psychopathological Manifestations in Schizophrenic Patients: A Single-Center Study from Ecuador." This letter incorporates responses to both the reviewers' comments and the additional journal requirements outlined in your correspondence.

**Response to Reviewers' Comments:**

**Reviewer #1 Comments:**

*1. The Rosado et el. manuscript describes very good the relation between Toxoplasma gondii infection and Psychopathological manifestation in Schizophrenic patients.*

*The language used is very correct and clearly used. The title is very well choosen and the abstract very good written. The statistical analysis were very performed. The conclusion are very good build.*

**Response**: We are grateful for your positive feedback. We have maintained the manuscript's clarity and further refined it according to the journal requirements and Reviewer 1’s comments to better convey our paper.

**Reviewer #2 Comments:**

*1. The references exhibit inconsistency in formatting and fail to adhere to a unified citation style also lack recent scholarly sources, notably missing key studies from the past five years.*

*2. The disconnect between the references cited within the text and their relevance to both the introduction and discussion sections of the article creates a significant discrepancy, undermining the coherence and integrity of the paper's argumentation.*

**Responses:**

1. We have updated the references to include key studies from the past five years (now more than 50% of references are from the past five years), ensuring our research is grounded in the most current and relevant scientific literature.
2. We have revised the entire list of references for consistency with PLOS ONE's citation style and included recent studies to enhance the manuscript's scholarly depth.

**Responses to Journal Requirements:**

***Requirement 1: Manuscript Style and File Naming***

*1. Please ensure that your manuscript meets PLOS ONE's style requirements, including those for file naming. The PLOS ONE style templates can be found at*

*https://journals.plos.org/plosone/s/file?id=wjVg/PLOSOne_formatting_sample_main_body.pdf and https://journals.plos.org/plosone/s/file?id=ba62/PLOSOne_formatting_sample_title_authors_affiliations.pdf*

**Response**: We have reviewed and applied the PLOS ONE style templates to our manuscript, ensuring full compliance with the formatting guidelines and file naming conventions.

***Requirements 2 & 3: Data Depositing for Enhanced Citation and Accessibility***

*2. Note from Emily Chenette, Editor in Chief of PLOS ONE, and Iain Hrynaszkiewicz, Director of Open Research Solutions at PLOS: Did you know that depositing data in a repository is associated with up to a 25% citation advantage (https://doi.org/10.1371/journal.pone.0230416)? If you’ve not already done so, consider depositing your raw data in a repository to ensure your work is read, appreciated and cited by the largest possible audience. You’ll also earn an Accessible Data icon on your published paper if you deposit your data in any participating repository (https://plos.org/open-science/open-data/#accessible-data).*

*3. In the online submission form, you indicated that [The data underlying the results can be fully provided upon request.].*

*All PLOS journals now require all data underlying the findings described in their manuscript to be freely available to other researchers, either 1. In a public repository, 2. Within the manuscript itself, or 3. Uploaded as supplementary information.*

*This policy applies to all data except where public deposition would breach compliance with the protocol approved by your research ethics board. If your data cannot be made publicly available for ethical or legal reasons (e.g., public availability would compromise patient privacy), please explain your reasons on resubmission and your exemption request will be escalated for approval.*

**Response**: In alignment with the journal's policy and to enhance citation potential, we have deposited our de-identified dataset in the Open Science Framework (OSF) repository. This action ensures our data is accessible and supports the integrity and reach of our work.

***Requirement 4: Placement of Ethics Statement***

*4. Your ethics statement should only appear in the Methods section of your manuscript. If your ethics statement is written in any section besides the Methods, please delete it from any other section.*

**Response**: The ethics statement is now appropriately located solely in the Methods section of our manuscript, ensuring clarity and adherence to ethical guidelines.

In conclusion, we hope the revisions and responses provided address the concerns raised by the reviewers and meet the journal's requirements. We believe these changes significantly improve our manuscript and demonstrate our commitment to contributing valuable, accessible, and ethically sound research to the scientific community.

We appreciate the opportunity to refine our work and look forward to the possibility of our manuscript being published in PLOS ONE.

Thank you for considering our revised manuscript.

Kind regards,

Dr. Diego Rosado
